# Anti-Fatigue Effects of *Lycium barbarum* Polysaccharide and Effervescent Tablets by Regulating Oxidative Stress and Energy Metabolism in Rats

**DOI:** 10.3390/ijms231810920

**Published:** 2022-09-18

**Authors:** Yanfeng Peng, Linlin Zhao, Ke Hu, Yongjing Yang, Jin Ma, Yuqing Zhai, Yan Jiang, Dejun Zhang

**Affiliations:** 1 College of Eco-Environmental Engineering, Qinghai University, Xining 810016, China; 2 College of Medical, Qinghai University, Xining 810016, China; 3 College of Medical, Shanghai University, Shanghai 200444, China

**Keywords:** *Lycium barbarum* polysaccharide, effervescent tablets, fatigue, biomarkers, oxidative stress, energy metabolism

## Abstract

The purpose of this study was to investigate the anti-fatigue effect of natural *Lycium barbarum* polysaccharide (LBP) during exercise, develop a functional anti-fatigue effervescent tablet by applying LBP to practical products, and help patients who have difficulty swallowing conventional tablets or capsules. LBP was extracted with water, and DEAE-52 cellulose was used for purification. The chemical structure and monosaccharide composition of LBP by Fourier transform infrared spectroscopy (FI-IR) and ion chromatography (IC). *Lycium barbarum* polysaccharide effervescent tablets (LBPT) were prepared by mixing LBP and an excipient. Animal experiments showed that LBP and LBPT significantly increased the exhaustive swimming time in rats. LBP and LBPT improved biochemical markers in rat serum, such as lactic acid and creatine kinase, enhanced the antioxidant capacity of rat muscle, and reversed the decrease in serum glucose, ATP and glycogen content caused by exercise. Transmission electron microscopy showed that LBP and LBPT increased the density of mitochondria in rat liver. In addition, molecular experiments showed that LBP and LBPT could improve oxidative stress caused by exercise by regulating the Nrf2/HO-1 signaling pathway and regulating energy metabolism via the AMPK/PGC-1α signaling pathway.

## 1. Introduction

Fatigue, usually defined as a physical inability to maintain a specific level of energy or the overall inability to maintain predetermined exercise intensity, is a temporary reduction in working capacity and physical function due to increased energy consumption in various organs caused by physical activity [1,2,3]. Fatigue often leads to lower motor function, lower work efficiency and more accidents. Long-term fatigue often increases the incidence of other diseases, such as multiple sclerosis, Parkinson’s disease and depression, which seriously endanger the work and life of patients [4,5,6,7,8].

Fatigue is strongly correlated with high-intensity exercise. In high-intensity exercise, the body produces metabolites, such as lactic acid and urea nitrogen. The accumulation of these metabolites changes the microenvironment in the body, leading to intracellular acidosis, further affecting muscle function, and thus causing fatigue [9]. Severe exercise can lead to the production and accumulation of free radicals, and rapidly consume energy substances, such as glucose, liver glycogen and muscle glycogen. The reduction in energy substances and oxidative stress caused by free radical accumulation are the key factors of peripheral fatigue [10,11]. Currently, effective drugs for fatigue treatment, such as some antidepressants and central nervous system stimulants, usually bring certain harm to cardiovascular, cerebrovascular and nervous systems and are prone to drug dependence [12]. Therefore, natural anti-fatigue substances without side effects are increasingly sought, in order to improve human exercise capacity and reduce fatigue.

The Qinghai–Tibet Plateau has a natural environment located at a high altitude with a low oxygen concentration, strong sunlight and large temperature differences. In this environment, it is easy to fatigue. Therefore, in the Tibetan medicine system, native plants are extensively used to alleviate fatigue symptoms [13]. *Lycium barbarum* (Goji berry) is the mature fruit of *Lycium barbarum* L., a shrub of the *Solanaceae* family. *Lycium barbarum* L. mostly blossoms from July to August each year and bears fruit every September; it is harvested in late September [14] and has been used as a traditional Tibetan medicine to relieve fatigue for thousands of years [15]. The reported chemical constituents of *Lycium barbarum* include polysaccharides, polyphenols, flavonoids, lignans, alkaloids, terpenoids, etc. [16]. LBP is a water-soluble polymer, mainly containing arabinose, glucose, galactose, mannose, rhamnose, xylose, etc. LBP is one of the main active ingredients of *Lycium barbarum* [17]. Modern pharmacological studies have shown that LBP has a variety of functions, including antioxidant, anti-aging, anti-tumor, immune regulation, hypoglycemic and hypolipidemic effects [18]. However, previous studies on the anti-fatigue mechanism of LBP are lacking.

In this study, LBP was isolated from *Lycium barbarum*. After purification, the chemical structure and monosaccharide composition of LBP was analyzed by FI-IR and IC. Moreover, a functional anti-fatigue effervescent tablet was developed by combining LBP with an excipient [19]. The fatigue model induced by forced swimming in rats was used to study the anti-fatigue effect of LBP and its related potential mechanism using a *Rhodiola Crenulata* capsule [20] as a positive control and LBPT as a parallel control. The application of LBP in effervescent tablets will be suitable for fatigue patients, and lay the foundation for the efficient use of LBP in the future.

## 2. Results

### 2.1. FT-IR Spectrum

**Figure 1:**
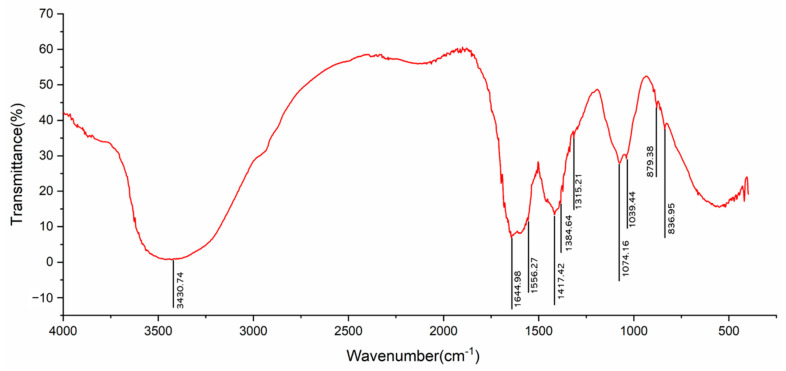
Infrared spectrum of LBP.

The results show that the absorption band at 3600–3200 cm^−1^ is the stretching vibration absorption peak of -OH, and the absorption peak in this region is the characteristic peak of saccharide (Figure 1) [21]. There is an absorption peak at 1644 cm^−1^, which is related to the asymmetric stretching vibration of C=O [22]. There is an absorption peak at 1556 cm^−1^, which is attributed to the C=O stretching vibration and an absorption peak at 1417 cm^−1^, which is the C-O stretching vibration [23]. The absorption peaks at 1384 cm^−1^ and 1315 cm^−1^ are attributed to the C=O symmetric stretching vibration [24], and there are absorption peaks at 1074 cm^−1^ and 1039 cm^−1^, which are attributed to the stretching vibration of the C-O-H and C-O-C group [25,26]. The absorption peak at 879 cm^−1^ is related to the C-H an-gular vibration of the equatorial bond other than the C-H isomerization of the terminal group of the pyran ring; it can be concluded that LBPs mainly contain pyranose, such as galactose, glucose and α-D-arabinose derivatives [27,28]. The absorption peak at 836 cm^−1^ is related to the C-H angular vibration of the β-terminal group difference of pyran ring. Therefore, it can be inferred that β-D-galactopyran may be the main component of LBP [29].

### 2.2. Monosaccharide Composition Analysis

**Figure 2:**
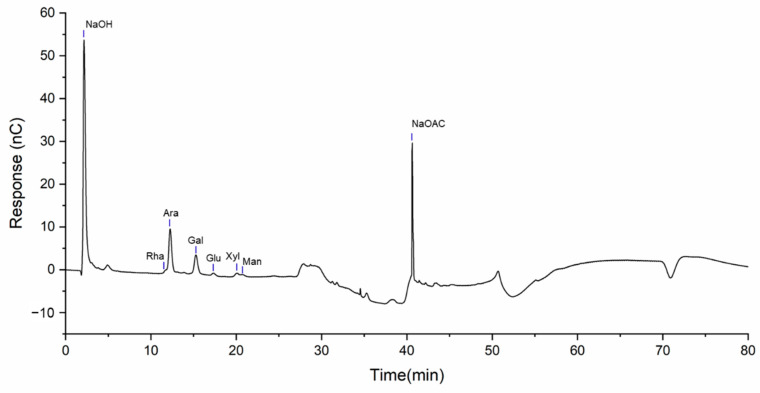
Ion chromatogram of LBP.

The monosaccharide composition of LBP was further revealed by ion chromatography, and the results were compared with those of the standard substance (Figure S1). The ion chromatogram shows that the characteristic peak of sodium hydroxide appeared at 2.0 min, the characteristic peak of rhamnose (Rha) was detected at 11.65 min, the characteristic peak of arabinose (Ara) was found at 12.19 min, the characteristic peak of galactose (Gal) was observed at 15.19 min, the characteristic peak of glucose (Glu) was detected at 17.26 min, the characteristic peak of xylose (Xyl) was seen at 19.97 min, the characteristic peak of mannose (Man) was recorded at 20.57 min, and the characteristic peak of sodium acetate was detected at 41 min (Figure 2).

Therefore, we infer that LBP is composed of Rha, Ara, Gal, Glu, Xyl and Man with a molar ratio of 1.00:11.35:6.10:0.56:1.08:0.71. Combined with the infrared spectroscopy results, we found that α-D-arabinose and β-D-galactopyranose may be the main components of LBP.

### 2.3. Effects of LBP and LBPT on Body Weight and Organ Index in Rats

**Figure 3:**
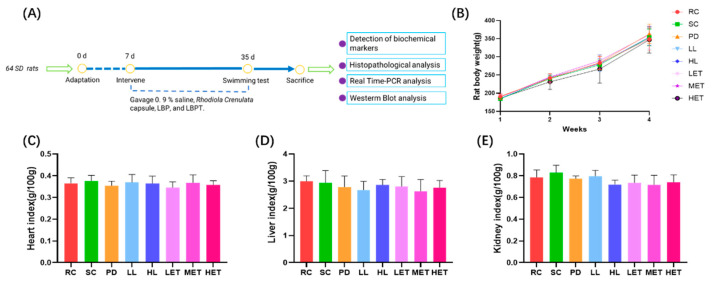
Effects of LBP and LBPT on body weight and organ index. (**A**) The experiment scheme, (**B**) rats’ body weight, (**C**) heart index, (**D**) liver index, (**E**) kidney index. Data are expressed as mean ± SD, N = 8.

By recording the body weight of rats, it was found that there was no significant difference in body weight between the intervention group and the SC group after 28 days of intervention (Figure 3B). The hearts, livers, and kidneys of the rats in each group were weighed to evaluate the organ index. The results show that the treatment of LBP and LBPT did not significantly change the organ index of the heart, liver, and kidney (Figure 3C–E). Therefore, LBP and LBPT also did not significantly change the body weight and organ index of the main organs in rats.

### 2.4. Effects of LBP and LBPT on Body Weight and Organ Index in Rats

**Figure 4:**
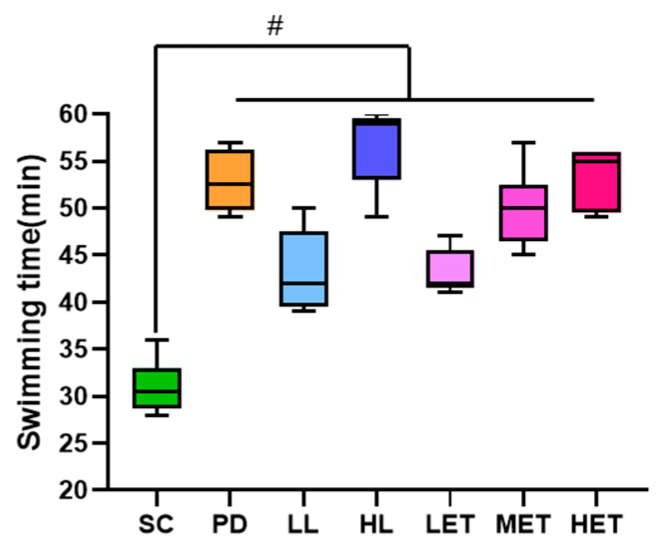
Effects of LBP and LBPT on exhaustive swimming time of rats. Data are expressed as mean ± SD, N = 8. #: vs. SC, *p* < 0.05.

An exhaustive swimming test is a commonly used anti-fatigue evaluation method that is applied in many anti-fatigue studies. In exhaustive swimming tests, a prolonged swimming time indicates increased exercise capacity and reduced fatigue [30,31]. Compared with the SC group, the exhaustive swimming time of each dose of LBP and LBPT treatment group was longer, and the difference was statistically significant (*p* < 0.05) (Figure 4); these results suggest that LBP and LBPT have significant anti-fatigue activity and can increase exercise tolerance in a dose-dependent manner.

### 2.5. Biochemical Parameters of LBP and LBPT in Improving Exercise Fatigue

#### 2.5.1. The Effects of LBP and LBPT on Serum Biochemical Parameters in Fatigue Rats

**Figure 5:**
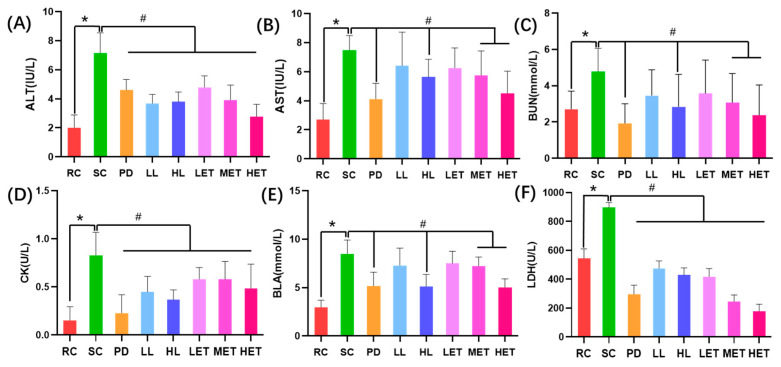
The effects of LBP and LBPT on serum biochemical parameters. (**A**) ALT, (**B**) AST, (**C**) BUN, (**D**) CK, (**E**) BLA, (**F**) LDH. Data are expressed as mean ± SD, N = 8, *: vs. RC *p* < 0.05, #: vs. SC, *p* < 0.05.

A variety of biochemical indicators have been used to assess fatigue after exercise, such as CK, AST, ALT, LDH, BUN and BLA [32,33]. Compared with the RC group, the activities of ALT, CK and LDH in the serum of rats were significantly increased after the swimming test (*p* < 0.05), and the levels of ALT, CK and LDH after exercise were significantly decreased after treatment with LBP and LBPT (*p* < 0.05) (Figure 5A,D,F). Similar trends were also found in AST, BUN and BLA indexes. Severe exercise led to AST activity in serum, and BUN and BLA contents significantly increased (*p* < 0.05); however, these significantly decreased in HL, MET and HET groups (*p* < 0.05) (Figure 5B,C,E). The above results show that intense exercise can cause the body to produce and accumulate many metabolites, thereby changing the homeostasis of the body’s internal environment. LBP and LBPT could improve the biochemical parameters related to exercise fatigue after intense exercise, thereby reducing the harm of fatigue.

#### 2.5.2. Regulation of LBP and LBPT on Muscle Tissue Oxidative Stress in Fatigue Rats

**Figure 6:**
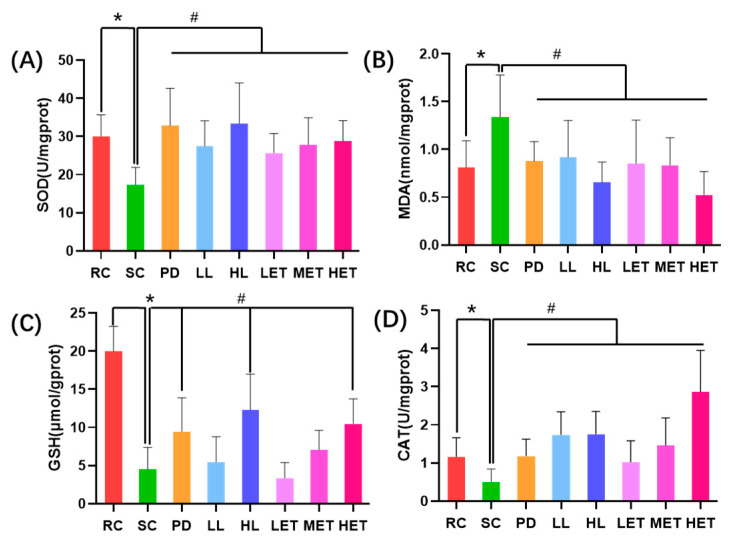
The effects of LBP and LBPT on oxidative stress parameters. (**A**) SOD, (**B**) MDA, (**C**) GSH, (**D**) CAT. Data are expressed as mean ± SD, N = 8, *: vs. RC *p* < 0.05, #: vs. SC, *p* < 0.05.

During exercise, oxidative stress could lead to muscle damage and fatigue [34]. Therefore, we studied the effects of LBP and LBPT on muscle oxidative stress in rats and detected oxidative stress-related indicators, including CAT, MDA, GSH and SOD. Compared with the RC group, the SOD level in the muscle of rats after intense exercise was significantly decreased, while the MDA level was significantly increased (*p* < 0.05); this state was reversed by large-strain LBP and LBPT (*p* < 0.05) (Figure 6A,B). After exercise, the GSH content in the muscle of rats significantly decreased (*p* < 0.05), but increased after the treatment with LBP and LBPT (*p* < 0.05) (Figure 6C). Similar to the change in GSH content, CAT activity significantly decreased after exercise, but increased in LBP and LBPT treatment groups (*p* < 0.05) (Figure 6D). The above results show that, during exercise, the phenomenon of oxidative stress in muscle cells will be intensified, thereby damaging cells. LBP and LBPT can improve oxidative muscle stress after strenuous exercise, thereby reducing muscle fatigue.

#### 2.5.3. Regulation of LBP and LBPT on Energy Metabolism in Fatigue Rats

**Figure 7:**
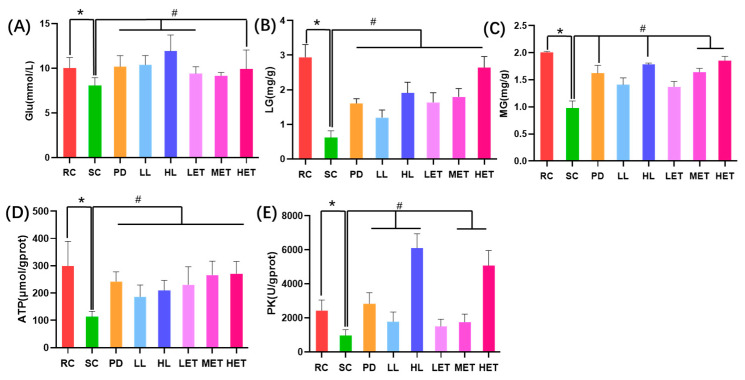
Regulation by LBP and LBPT of energy metabolism. (**A**) blood Glu; (**B**) liver glycogen contents, LG; (**C**) muscle glycogen contents, MG; (**D**) muscle ATP; and (**E**) Muscle PK. Data are expressed as mean ± SD, N = 8, *: vs. RC *p* < 0.05, #: vs. SC, *p* < 0.05.

The consumption of energy storage may also lead to exercise fatigue, and the levels of blood glucose, liver glycogen and muscle glycogen are one of the important criteria for evaluating fatigue [35]. Compared with the RC group, the blood glucose level of the SC group significantly decreased after exercise (*p* < 0.05), while the blood glucose level of the LBP and LBPT groups significantly increased (*p* < 0.05) (Figure 7A). According to the trend of serum glucose level, the glycogen content in muscle and liver after exercise also significantly decreased (*p* < 0.05). However, compared with the SC group, the glycogen content in LBP and LBPT groups significantly increased (*p* < 0.05) (Figure 7B,C).

In order to further study the effects of LBP and LBPT on the main processes of energy metabolism, we compared the levels of PK and ATP in the muscles of rats. Intensive exercise significantly decreased the ATP content in muscle (*p* < 0.05) (Figure 7D) and decreased PK activity in muscle (Figure 7E). The treatment of LBP and LBPT could effectively reverse this state (*p* < 0.05). The data show that exercise can lead to the rapid consumption of energy substances and deteriorate the normal energy metabolism cycle of the body. The intervention of LBP and LBPT can effectively regulate the imbalance of energy metabolism caused by exercise and supplement the energy lost by exercise faster.

#### 2.5.4. Correlation Analysis and PCA Analysis of Biochemical Parameters

**Figure 8:**
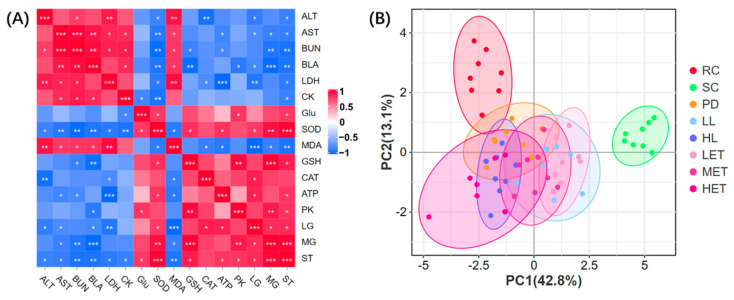
Biochemical parameters correlation analysis and PCA analysis. (**A**) Correlation analysis, (**B**) PCA analysis. N = 8, *: *p* < 0.05, **: *p* < 0.01, ***: *p* < 0.001. The confidence level of the confidence ellipse in the PCA analysis is 95%.

To study the contribution of biochemical parameters to exhaustive swimming time (ST). We analyzed the correlation between each biochemical parameter and exhaustive swimming time. The results show that exhaustive swimming time was significantly positively correlated with muscle glycogen, liver glycogen, PK, ATP, GSH, SOD and Glu levels. The levels of ALT, AST, BUN, BLA, LDH, CK and MDA were significantly negatively correlated with swimming time (Figure 8A). Thus, we can conclude that improving energy metabolism and oxidative stress factors can effectively enhance the exercise capacity of rats. Furthermore, we found that the ability of the body to deal with metabolites was significantly correlated with the ability to pass energy metabolism and oxidative stress. For example, BUN was significantly negatively correlated with muscle glycogen and ATP, but was significantly positively correlated with MDA (Figure 8A); this suggests that LBP and LBPT may improve the microenvironment of fatigue rats by regulating energy metabolism and oxidative stress; therefore, it can play an anti-fatigue role and improve the exercise ability of rats.

In order to further study the overall effect of LBP and LBPT on fatigue rats, 15 biochemical parameters were analyzed by principal component analysis (PCA). The data show that the SC group clustered farther from the RC group, and the difference between the two groups was significant. Compared with the SC group, the confidence ellipses of PD, LL, LH, LET, MET and HET groups were not intersected with the SC group, indicating that each drug group was significantly different from the SC group. The confidence ellipses of PD, HL and HET groups intersected with the RC group, suggesting that the PD, HL and HET groups showed little difference compared to RC in 15 biochemical parameters (Figure 8B). Therefore, we believe that severe exercise can lead to changes in many biochemical parameters in rats, and the intervention of LBP and LBPT can effectively reverse fatigue, among which PD, HL and HET groups have better effects.

Furthermore, we found that the LL group and LET group, HL group and HET group have more confidence ellipse overlap (Figure 8B), which suggests that the similarity between these groups is high, indicating that the treatment of LBPT may not affect the efficacy of LBP. We also found that the confidence ellipses of LET, MET and HET showed a progressive movement, suggesting that the efficacy of LBPT may be dose-dependent.

### 2.6. Histopathological Analysis

**Figure 9:**
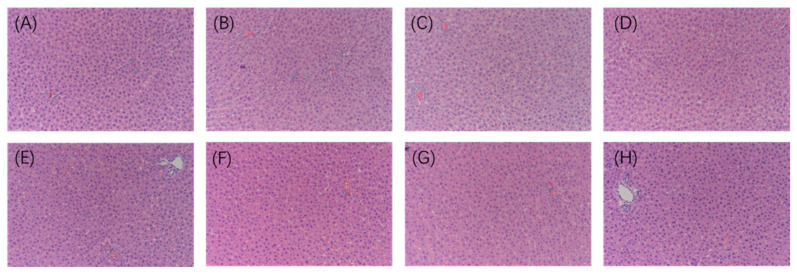
Histopathological analysis of liver tissue. (**A**) RC, (**B**) SC, (**C**) PD, (**D**) LL, (**E**) HL, (**F**) LET, (**G**) MET, and (**H**) HET. N = 3, Magnification ×200.

A histopathological analysis of the liver was performed to understand the morphological damage in the tissue. The results show that, compared with the RC group, the morphology of hepatocytes in the SC group did not change after exercise, and the treatment of LBP and LBPT did not significantly change the arrangement of cells in the liver (Figure 9). Therefore, LBP and LBPT treatment did not significantly change liver histopathology in rats, suggesting that LBP and LBPT have low side effects.

### 2.7. Transmission Electron Microscopy Analysis

**Figure 10:**
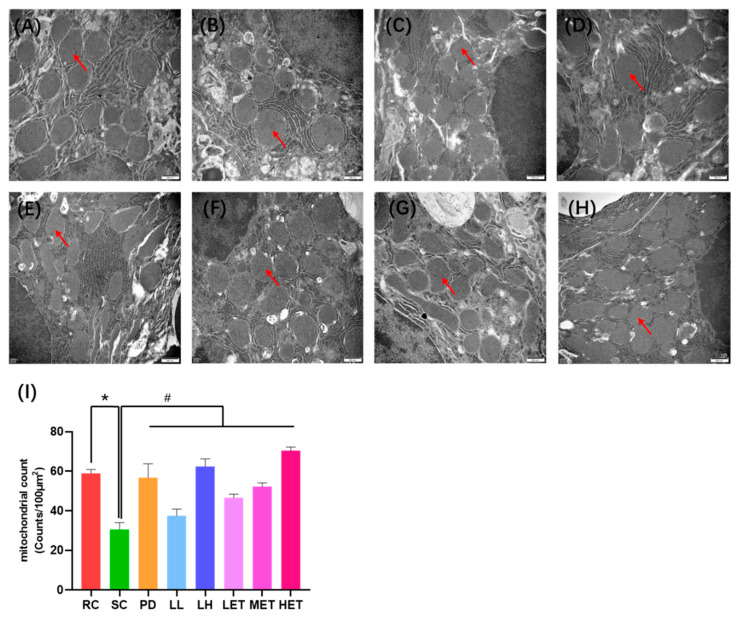
Transmission electron microscopy analysis of hepatocytes. (**A**) RC, (**B**) SC, (**C**) PD, (**D**) LL, (**E**) HL, (**F**) LET, (**G**) MET, (**H**) HET, (**I**) Mitochondrial count. Data are expressed as mean ± SD, N = 3, Magnification ×30,000. *: vs. RC *p* < 0.05, #: vs. SC, *p* < 0.05. Mitochondria are shown by red arrows.

In order to further understand the effect of fatigue on the morphology of hepatocytes, we analyzed the cells using transmission electron microscopy (Figure 10A–H). The results show that, compared with the RC group, the mitochondrial density in hepatocytes significantly decreased after strenuous exercise (*p* < 0.05), while the mitochondrial density in hepatocytes significantly increased after the treatment of LBP and LBPT (*p* < 0.05) (Figure 10I). Therefore, we found that intense exercise can lead to mitochondrial fusion, thereby increasing energy metabolism. However, this phenomenon can lead to the deterioration of the mitochondrial fusion/division balance, resulting in cell death [36,37,38]. LBP and LBPT can effectively increase the division of mitochondria, thereby increasing the output of cell energy, preventing mitochondrial fusion, and thus protecting cells from injury caused by exercise.

### 2.8. Molecular Mechanism of LBP and LBPT Regulating Oxidative Stress in Muscle Tissue of Fatigue Rats

**Figure 11:**
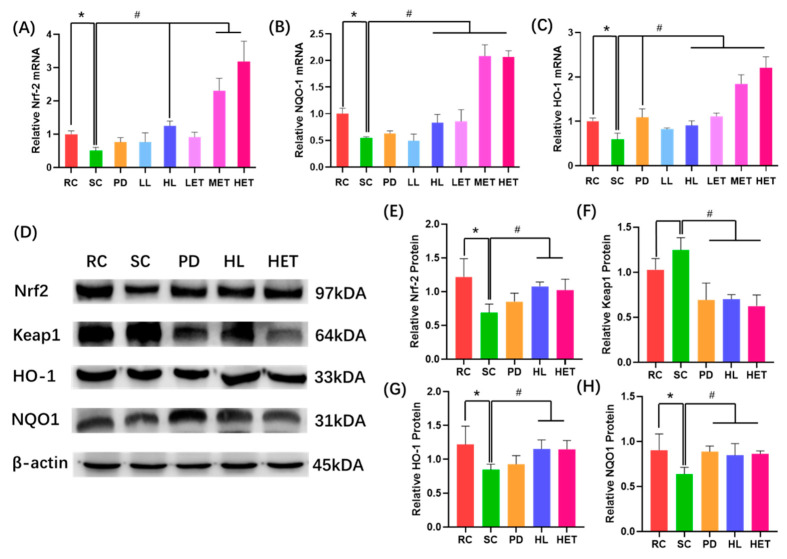
Molecular mechanism of LBP and LBPT regulating oxidative stress in muscle tissue of fatigue rats. (**A**) Relative expression of muscle Nrf2 mRNA, (**B**) relative expression of muscle NQO1 mRNA, (**C**) relative expression of muscle HO-1 mRNA, (**D**) expression of Nrf2/HO-1 pathway-related proteins, (**E**) relative expression of muscle Nrf2 protein, (**F**) relative expression of muscle Keap1 protein, (**G**) relative expression of muscle HO-1 protein, and (**H**) relative expression of muscle NQO1 protein. Data are expressed as mean ± SD, N = 3. *: vs. RC *p* < 0.05, #: vs. SC, *p* < 0.05.

In order to further clarify the molecular mechanism of LBP and LBPT on oxidative stress, RT-PCR and Western blotting were used to study the effects of LBP and LBPT on the expression patterns of some important antioxidant signaling pathways. The RT-PCR results show that, compared with the RC group, the swimming test significantly reduced the mRNA relative expression of Nrf2, HO-1 and NQO1. The treatment of LBP and LBPT significantly increased the expression levels of Nrf2, HO-1 and NQO1 mRNA (*p* < 0.05) (Figure 11A–C). After LBP and LBPT treatment, the protein levels of Nrf2, HO-1 and NQO1 in the muscle of fatigue rats significantly increased, and the expression level of Keap1 in muscle significantly decreased (*p* < 0.05) (Figure 11D–H), indicating that LBP and LBPT activated Nrf2 and increased the protein expressions of HO-1 and NQO1 by the cascade effect. Previous biochemical analysis of the Nrf2 downstream pathway also show that the treatment of LBP and LBPT increased the levels of SOD and CAT in muscle, and reduced the accumulation of MDA in muscle. In conclusion, these results suggest that LBP and LBPT can regulate oxidative stress through the Nrf2/HO-1 signaling pathway, thus playing an anti-fatigue role during exercise.

### 2.9. Molecular Mechanism of LBP and LBPT Regulating Energy Metabolism in Muscle Tissue of Fatigue Rats

**Figure 12:**
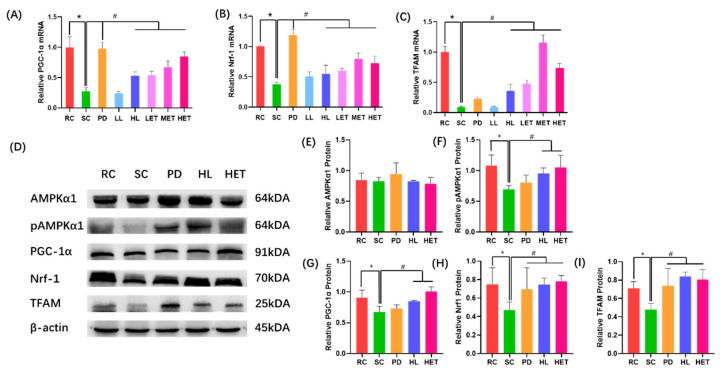
Molecular mechanism of LBP and LBPT regulating energy metabolism in muscle tissue of fatigue rats. (**A**) Relative expression of muscle PGC-1α mRNA, (**B**) relative expression of muscle Nrf1 mRNA, (**C**) relative expression of muscle TFAM mRNA, (**D**) expression of AMPK/PGC-1α pathway-related proteins, (**E**) relative expression of muscle AMPKα1 protein, (**F**) relative expression of muscle pAMPKα1 protein, (**G**) relative expression of muscle PGC-1α protein, (**H**) relative expression of muscle Nrf1 protein, and (**I**) relative expression of muscle TFAM protein. Data are expressed as mean ± SD, N = 3. *: vs. RC *p* < 0.05, #: vs. SC, *p* < 0.05.

Our results show that strenuous exercise significantly reduced the relative mRNA expression of PGC-1α, Nrf1 and TFAM. The treatment of LBP and LBPT effectively reversed this phenomenon (*p* < 0.05) (Figure 12A–C). After treatment with LBP and LBPT, the protein levels of AMPKα1 in the muscle of fatigued rats did not significantly change, but the expression levels of pAMPKα1, PGC-1α, Nrf1 and TFAM proteins in muscle increased significantly (*p* < 0.05) (Figure 12D–I); this indicated that LBP and LBPT activated AMPKα1. AMPKα1 protein increased the protein expression of PGC-1α, Nrf1 and TFAM by phosphorylation, thereby promoting mitochondrial division and increasing energy metabolism. Therefore, these results suggest that polysaccharide and effervescent tablets can regulate energy metabolism through the AMPK/PGC-1α signaling pathway, thus playing an anti-fatigue role during exercise.

## 3. Discussion

The aim of this study was to find a natural anti-fatigue dietary supplement and apply it to effervescent tablets to provide an alternative for patients with dysphagia. To achieve this, LBP was isolated from *Lycium barbarum*. After purification, the chemical structure and monosaccharide composition of LBP was analyzed by infrared spectroscopy and ion chromatography; it was found that LBP are mainly composed of α-D-arabinose and β-D-galactose. Animal experiments found that LBP and its effervescent tablets can significantly alleviate exercise-induced fatigue. LBP and LBPT significantly prolonged the exhaustive swimming time of SD rats in the swimming test. Additionally, the enhanced exercise-fatigue- and injury-related biochemical parameters (ALT, AST, BLA, BUN, CK, and LDH) improve both antioxidant capacity (SOD, CAT, GSH and MDA) and muscle energy metabolism (Glu, MG, LG, ATP and PK). We also found that LBP and LBPT did not cause damage to liver tissue, and further transmission electron microscopy found that LBP and LBPT increased mitochondrial density in hepatocytes. We also proved that LBP and LBPT could improve exercise fatigue by Nrf2/HO-1 signal-related antioxidant defense and AMPK/PGC-1α signal-related regulatory energy metabolism; these results suggest that LBP may be a potential and natural anti-fatigue dietary supplement candidate, and the effervescent tablets made from LBP have similar pharmacological activities.

A combination of multiple biomarkers has been used to assess exercise-induced fatigue [39]. In this study, we evaluated several biomarkers. Exhausted swimming time in rats can be used as a biomarker reflecting endurance and fatigue [40]. LBP and LBPT prolonged the exhaustive swimming time of rats, especially in HL and HET groups, indicating that the intervention of LBP and LBPT can effectively improve the exercise endurance of rats. We further examined the wet biomarkers from blood and tissues. During intense exercise, the main energy supply mode of the body is anaerobic glycolysis or glycogen decomposition, which produces lactic acid, and the accumulation of lactic acid is one of the main factors leading to body fatigue [41]. After a forced swimming test, the level of lactic acid in rat serum significantly increased but decreased in the LBP and LBPT treatment groups. The decrease in LDH activity after LBP and LBPT treatment may be caused by the feedback from the decrease in lactic acid level. CK, another important biomarker, is one of the indicators of muscle injury. Intensive exercise can damage the structure of skeletal muscle cells, resulting in an increase in CK level [42]. Our study found that intense exercise increased serum CK levels in rats, while LBP and LBPT significantly decreased serum CK levels in rats. During exercise, the liver is damaged by microenvironment changes and oxidative stress; this injury is usually accompanied by elevated levels of AST and ALT [43]. The levels of AST and ALT in the serum of rats treated by the swimming test increased, which could be reversed by LBP and LBPT. From the results of serum biomarkers, we concluded that LBP could significantly alleviate the biochemical parameters related to exercise fatigue and injury after acute exercise. In addition to the above biomarkers, other biomarkers, such as nitric oxide synthase, myoglobin, inflammatory markers (blood interleukin and interleukin) have also been used in recent years [44]. Therefore, these biomarkers related to fatigue need to be further studied to clarify the anti-fatigue effect of LBP in future research.

Oxidative stress during strenuous exercise may lead not only to exercise-induced fatigue, but also to muscle damage [45]. During exercise, reactive oxygen species (ROS) will be generated, resulting in the oxidation of proteins, lipids or nucleic acids, which further aggravates oxidative stress [46]. Therefore, we used MDA, GSH, CAT and SOD indicators to examine the oxidative stress levels in different groups. The levels of SOD, GSH and CAT in the muscle of rats treated with LBP and LBPT significantly increased, while the level of MDA significantly decreased. A correlation analysis also found that exhaustive swimming time was significantly correlated with MDA, GSH and SOD; this indicated that the treatment of LBP and LBPT significantly reduced oxidative stress during acute exercise. We further studied the molecular mechanism of LBP and LBPT involved in antioxidants. Nrf2 is a key factor in regulating cellular antioxidant response [47]. Under the stimulation of ROS, Nrf2 is separated from Keap1 protein and translocated to the nucleus to bind to ARE and regulate the expression of downstream genes involved in cellular antioxidant responses, such as HO-1 and NQO1 [48,49]. RT-PCR analysis showed that Nrf2 mRNA expression was increased after treatment with LBP and LBPT, and HO-1 and NQO1 mRNA expression downstream was also increased. WB analysis showed that the intervention of LBP and LBPT significantly increased the expression of Nrf2 protein, reduced the expression of Keap1 protein, and increased the expression of HO-1 and NQO1 proteins downstream; this indicates that LBP and LBPT achieve an antioxidant effect through the Nrf2/HO-1 pathway.

Intensive exercise consumes a lot of energy substances, and this rapid consumption will lead to an imbalance of energy metabolism in the body, resulting in decreased exercise capacity [50]. Our study found that swimming experiments could significantly consume ATP and blood glucose, and reduce the content of liver glycogen and muscle glycogen. The contents of ATP and blood glucose in the muscle of rats treated with LBP and LBPT significantly increased, and the contents of liver glycogen and muscle glycogen also showed similar trends; this shows that the intervention of LBP and LBPT can significantly increase the content of energy substances. PK is one of the key factors affecting ATP regeneration [51]. Severe exercise significantly decreased the activity of PK in rat muscles, which could be reversed by LBP and effervescent tablets. Therefore, the anti-fatigue effect of LBP and LBPT seems to be related to regulating energy metabolism in rats, which was corroborated by the use of transmission electron microscopy for hepatocytes. The intervention of LBP and effervescent tablets can effectively increase the density of mitochondria, thereby increasing the energy metabolism of cells. AMPK/PGC-1α signaling pathway is one of the main signaling pathways that regulates mitochondrial fission. When ATP level decreases in vivo, AMPKα1 protein activates PGC-1α through phosphorylation, thereby activating Nrf1 and TFAM downstream, regulating mitochondrial fission and improving the level of energy metabolism in the body [52,53]. We further studied the molecular mechanism involved in energy metabolism. The RT-PCR results show that the expression of PGC-1α mRNA was increased, and the downstream Nrf1 and TFAM mRNA levels were increased after treatment with LBP and effervescent tablets. WB analysis showed that the intervention of LBP and effervescent tablets significantly increased the expression of pAMPKα1 protein, further improved the expression of PGC-1α protein, and increased the expressions of the Nrf1 and TFAM proteins downstream; this indicates that LBP and effervescent tablets regulate energy metabolism through AMPK/PGC-1α pathway.

We prepared LBPT to provide alternatives for patients with dysphagia. Furthermore, we carried out parallel experiments between LBP and LBPT, finding that LBP and LBPT had similar pharmacological effects. Our PCA analysis also proved this point. The confidence ranges of the same dose of LBP and LBPT were coincident, indicating that the preparation of LBPT did not affect the efficacy of LBP. However, we did not set the excipient group, so whether this excipient has the corresponding biological activity needs further exploration.

## 4. Materials and Methods

### 4.1. Rats and Reagents

Sixty-four male SD rats (4 weeks old; body weight 180–210 g) were provided by Chongqing Tengxin Biotechnology Co., Ltd. (Chongqing, China). All the animal care and use procedures were approved by the Medical Ethics Committee of Qinghai University School of Medicine (2019-1). *Lycium barbarum* was provided by Kangpu Biotechnology Co., Ltd. (Xining, China). *Rhodiola Crenulata* Capsule (Z20040023) was purchased from the First People’s Hospital of Qinghai Province (Xining, China). Blood urea nitrogen (BUN), blood lactic acid (BLA), lactate dehydrogenase (LDH), creatine kinase (CK), aspartate aminotransferase (AST), aspartate aminotransferase (AST), catalase (CAT), superoxide dismutase (SOD), malondialdehyde (MDA), glutathione (GSH), pyruvate kinase (PK), adenosine triphosphate (ATP), commercial blood glucose (Glu), and hematoxylin and eosin (H&E) kits were purchased from Nanjing Jiancheng Bioengineering Institute (Nanjing, China). BCA protein detection kits, muscle glycogen (MG) and liver glycogen (LG) detection kits are purchased from Elabscience Biotechnology Co., Ltd. (Wuhan, China). TRIzol, reverse transcription commercial kit and commercial SYBR green enzymes were provided by Tiangen Biochemical Technology Co., Ltd. (Beijing, China). RIPA cracking solution, TBST and 5% BSA blocking solution were provided by Solebo Technology Co., Ltd. (Beijing, China). The antibodies against Kelch-Like ECH Associated Protein 1 (Keap1), Nuclear Factor Erythroid 2-Related Factor 2 (Nrf2), Heme Oxygenase 1 (HO-1), NAD(P)H Dehydrogenase 1 (NQO1), 5′-AMP-Activated Protein Kinase Catalytic Subunit Alpha-1 (AMPKα1), Phosphorylated 5′-AMP-Activated Protein Kinase Catalytic Subunit Alpha-1 (pAMPKα1), Peroxisome Proliferator-Activated Receptor Gamma Coactivator 1-Alpha (PGC-1α), Nuclear Respiratory Factor 1 (Nrf1), Mitochondrial Transcription Factor 1 (TFAM), β-Actin and HRP Goat Anti-Rabbit IgG, 0.45 μm Nitrocellulose membrane and ECL luminescent solution were provided by ABclone Co., Ltd. (Wuhan, China). All other chemicals were purchased from Tianjin Damao Chemical Reagent Factory (Tianjin, China).

### 4.2. Method

#### 4.2.1. Preparation of LBP

The extraction of LBP was based on the previous method [54], and some modifications were made. *Lycium barbarum* was cleaned, dried, and crushed according to 1:20 (g/mL), and then, 90% ethanol was added at 70 °C for 2 h, followed by degreasing of the pigment. The residue was made in a ratio of 1:25 (g/mL) by adding distilled water at 90 °C for 4 h; filter supernatant was made in a ratio of 1:4 (V:V) by adding anhydrous ethanol and precipitating overnight. The precipitate was dissolved in a small amount of distilled water, and a sevage reagent (chloroform: n-butanol = 4:1) was added according to a ratio of 1:5 (V:V). The protein was separated by a liquid funnel and repeated three times. The residue was concentrated by rotary evaporation to allow viscosity and freeze-dried at −80 °C and 2.5 Pa for 1 d.

#### 4.2.2. Determination of LBP Content

The polysaccharide content was determined by a phenol–sulfuric acid method [55]. Standard curve preparation: 100 mg/mL glucose reference solution; 0.1, 0.2, 0.4, 0.8, and 1.0 mL were placed in test tube, supplemented with distilled water to 2.0 mL. After shaking with 1.0 mL 6% phenol, 5.0 mL concentrated sulfuric acid was rapidly dropped and immediately shaken. After being placed for 5 min, the mixture was heated in a boiling water bath for 15 min and cooled to room temperature (25 °C). The absorbance (A) was measured at 490 nm, and the standard curve was obtained with the mass concentration of glucose as the abscissa and the absorbance as the ordinate. The regression equation is Y = 6.3804X − 0.0279, R^2^ = 0.9992.

Determination of polysaccharide content: LBP was configured to 1 mg/mL aqueous solution, polysaccharide solution 2.0 mL, and determination of polysaccharide content. Each sample was subjected to three parallel experiments. The determination method is the same as above, using the standard curve to calculate the polysaccharide content.

The yield of polysaccharide is calculated as follows:

$$W=\frac{M_{t}}{M_{s}}\times C_{p}\times 100\%$$

*M_t_* is the quality of the total polysaccharide, *M_s_* is the quality of the sample, and *C_p_* is the content of polysaccharide.

The final yield of LBP was 4.73%.

#### 4.2.3. Purification of LBP

LBP was prepared into an aqueous solution with a mass concentration of 20 mg/mL, and a 30 mL crude polysaccharide solution was taken. The supernatant was separated by DEAE-52 cellulose column chromatography and eluted with distilled water. The elution flow rate was maintained at 1 mL/min. One tube was collected every 6 min, and the change in sugar content was detected by the phenol–sulfuric acid method [56]. The eluent was collected, and the polysaccharide content was detected by the phenol–sulfuric acid method after freeze-drying. The final purified polysaccharide content was 97.22%.

#### 4.2.4. FT-IR Spectroscopy

The chemical structure of LBP was analyzed by Fourier transform infrared spectroscopy [57]. Precisely weighed LBP (2 mg) and potassium bromide (200 mg) were pressed into tablets, and a blank control using potassium bromide powder was pressed into tablets; these tablets were separately placed in Fourier transform infrared spectrometer FT-IR650 (Tianjin Port East Technology Development Co., Ltd., Tianjin, China) scanning records.

#### 4.2.5. Monosaccharide Composition Analysis

The monosaccharide composition of LBP was determined by ion chromatography [58]. Then, 5 mg of LBP was precision weighed in ampoan ule bottle, 2 mL 3 mol/L trifluoroacetic acid as added, and the mixture was hydrolyzed at 120 °C for 3 h. The acid hydrolysis solution was transferred to the tube and dried by nitrogen blowing. Next, 5 mL of deionized water was added, dissolved and mixed. Then, 50 μL was added to 950 μL deionized water and centrifuged at 12,000 rpm for 5 min. The supernatant was analyzed by ion chromatography (Thermo Fisher, Shanghai, China). Chromatographic conditions: chromatographic column: Dionex Carbopac^TM^ PA20 (3 × 150 mm); mobile phase: A: H_2_O; B: 15 mM NaOH:100 mM NaOAC (1:1); flow rate: 0.3 mL/min; sample size: 5 μL; column temperature: 30 °C; detector: Electrochemical detector.

The results were compared with 16 monosaccharide standards (fucose (Fuc), aminogalactose hydrochloride (GalN), rhamnose (Rha), arabinose (Ara), glucosamine hydrochloride (GlcN), galactose (Gal), glucose (Glu), xylose (Xyl), mannose (Man), fructose (Fru), ribose (Rib), galactose aldehyde acid (GalA), glucuronic acid (GlcA), N-acetyl-D-glucosamine (GlcNAc), guluronic acid (GulA), and mannose aldehyde acid (ManA)) to determine the monosaccharide species and molar ratio.

#### 4.2.6. Preparation of LBPT

LBPT were composed of 20% LBP and 80% excipients (35% lactose, 3% sucralose, 2% ethanol, 20% citric acid and 20% sodium bicarbonate). After the powder raw materials were mixed evenly, wolfberry polysaccharide effervescent tablets were pressed in a single press.

#### 4.2.7. Animal Experiment

This study used 4-week-old male SD rats. Animals were maintained in a 12/12 h light/dark cycle, turned on at 7:00 a.m. and off at 7:00 p.m., with free access to normal food and water. After one week of adaptive culture, the rats were randomly divided into 8 groups (Figure 3A):

- Rest control group (RC, N = 8) received 0.9% saline by gavage;
- The swimming control group (SC, N = 8) received 0.9% normal saline by gavage;
- Positive drug group (PD, N = 8) received 54 mg/kg/d *Rhodiola Crenulata* capsules by gavage;
- LBP low-dose group (LL, N = 8) received 120 mg/kg/d LBP by gavage;
- LBP high-dose group (HL, N = 8) received 360 mg/kg/d LBP by gavage;
- LBPT low-dose group (LET, N = 8) received 600 mg/kg/d LBPT by gavage;
- LBPT middle-dose group (MET, N = 8) received 1200 mg/kg/d LBPT by gavage;
- LBPT high-dose group (HET, N = 8) received 1800 mg/kg/d LBPT by gavage;

After 28 consecutive days of daily drug intervention, groups (SC) to (HET) were assigned for forced swimming test, and rats in the group (SC) were used as non-swimming control. After the swimming experiment, rats in all groups were sacrificed, and blood, skeletal muscle, liver, heart and kidney were collected. Blood samples were centrifuged at 3500 rpm for 10 min at 4 °C to separate serum. All samples are stored at −80 °C for subsequent detection.

#### 4.2.8. Forced Swimming Test

Forced swimming tests were performed 1 h after the last drug intervention, as described earlier, with minor modifications [59]. Rats were placed separately in a swimming pool (150 × 280 × 280 cm) filled with water (25 ± 2 °C), and lead wires (5% of body weight) were connected to the tail roots of each rat. When uncoordinated movement was observed in each rat, and the rats failed to return to the water within 5 s, the exhaustive swimming time was recorded.

#### 4.2.9. Detection of Biochemical Markers in Rats

The activity of CK, AST, AST and AST, as well as the content of BUN, BLA and Glu in serum, were measured and calculated using certain kits. Liver tissue was homogenized with 0.9% saline, and LG content was determined and calculated using a kit. The muscle was homogenized with 0.9% saline, and the activities of CAT, SOD and PK, as well as the contents of MDA, GSH, ATP and MG, were measured and calculated with the kits.

#### 4.2.10. Histopathological Analysis of Rat Liver Tissue

A small part of the liver tissue was fixed with 10% neutral buffer formalin solution for 24 h, and then dehydrated by different grades of ethanol (80%, 95% and 100%), transparent in xylene and covered with paraffin. A 0.2 μm slice was then cut from paraffin-embedded tissue using a slicer (Leica Instruments GmbH, Wetzlar, Germany). The slides were prepared by dewaxing, stained with H&E, and sealed with neutral gum. Microphotographs were obtained using a Nikon DS-Ri2 microscope (Nikon Corporation, Tokyo, Japan).

Another part of the liver tissue was fixed with 2.5% glutaraldehyde and 4% paraformaldehyde at 4 °C for 2 h, rinsed with 0.1 M phosphate buffer, and then fixed with 1% osmic acid at 4 °C for 2 h. After rinsing with 0.1 M phosphate buffer, different grades of acetone (30%, 50%, 70%, 90% and 100%) were dehydrated. After embedding, sections were stained with uranium acetate and lead citrate, and finally observed using a JEM-1400 PLUS transmission electron microscope (Japan Electronics Corporation, Tokyo, Japan).

#### 4.2.11. RT-PCR Analysis of Rat Muscle Tissue

Total RNA was extracted from rat muscle tissue by using TRIzol and reverse transcribed. A Roche LightCycler 96 real-time fluorescence polymerase chain reaction system (Roche, Shanghai, China) was used for a quantitative real-time polymerase chain reaction, and amplification was carried out in 20 μL reaction volumes containing 1×SYBR Green. The relative expression of each gene was calculated compared with β-actin. The primers used for RT-PCR are shown in Table 1.

#### 4.2.12. Western Blot Analysis of Rat Muscle Tissue

Extract muscle protein using RIPA lysate. Protein concentration was determined by the BCA method. β-actin, Nrf2, KEAP1, NQO1, HO-1, AMPKα1, pAMPKα1, PGC1-1α, Nrf1 and TFAM proteins were isolated by SDS-PAGE gel electrophoresis system (WIX Technology Beijing Co., Ltd., Beijing, China). The protein was transferred to 0.45 μm NC membrane by an electro-transfer membrane system (Shanghai Peiqing Technology Co., Ltd., Shanghai, China) and blocked in 5% BSA for 1 h. The NC membrane was incubated with anti-β-actin (1:1000), Nrf2 (1:1000), Keap1 (1:1000), NQO1 (1:1000), HO-1 (1:1000). AMPKα1 (1:1000), p-AMPKα1 (1:1000), PGC-1α (1:1000), NRF1 (1:1000), TFAM (1:1000) were incubated overnight at 4 °C. After washing with 1×TBST, HRP Goat Anti-Rabbit IgG (1:4000) was incubated for 1 h at 25 °C. After washing with 1×TBST, ECL chemiluminescence solution was used for detection in an ODYSSEY Fc dual-color infrared laser imaging system (LI-COR, Lincoln, NE, USA).

#### 4.2.13. Statistical Analysis

The SPSS 17 software (IBM SPSS Modeler, Chicago, IL, USA) was used for statistical analysis of the results. Data were analyzed by one-way ANOVA to evaluate the difference between the two groups. A person correlation analysis was used to evaluate the correlation between different parameters. The heatmap and PCA graph was drawn by Omicshare (GeneDenovo Biotechnology Co Ltd., Guangzhou, China). The data are expressed as the mean ± standard deviation (mean ± SD). Results with * *p* < 0.05, ** *p* < 0.01, and *** *p* < 0.001 were considered significant.

## 5. Conclusions

**Figure 13:**
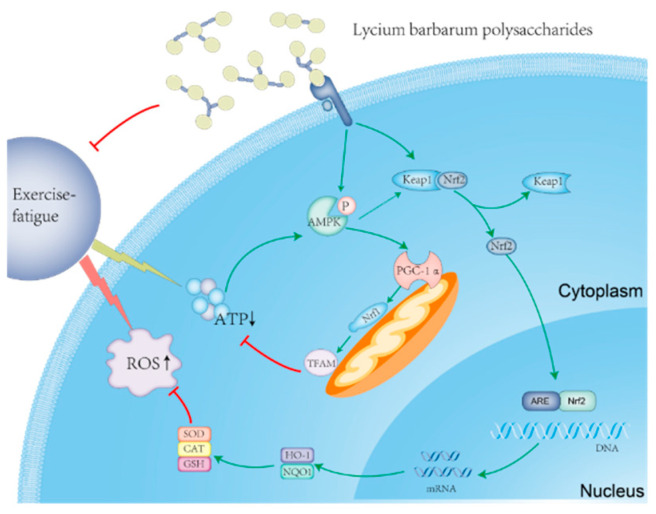
Anti-fatigue effect of LBP via the Nrf2 and PGC-1α signaling pathways in rats.

Fatigue caused by long-term exercise not only leads to a decline in exercise capacity, but may also be the cause of many diseases. Here, we proved that LBP can significantly alleviate fatigue caused by exercise. The beneficial effect of LBP on fatigue may be due to its positive effect on energy metabolism and antioxidant capacity (Figure 13); these findings suggest that LBP may be used as anti-fatigue dietary supplements for alleviating exercise-induced fatigue symptoms.

## Figures and Tables

**Table 1:** RT-PCR primer sequences.

Project	Forward (5′-3′)	Reverse (5′-3′)
Nrf2	CCTTCCTCTGCTGCCATTAGTC	GAACTCCACCGTGCCTTCAG
NQO1	GCCTACACGTATGCCACCAT	TGGACACCCTGCAGAGAGTA
HO-1	CTAAGACCGCCTTCCTGCTC	GCCTCTGGCGAAGAAACTCT
PGC1-1α	CGGTGGATGAAGACGGATTGCC	ATTGTAGCTGAGCTGAGTGTTGGC
Nrf1	TCTGCTGTGGCTGATGGAGAGG	GATGCTTGCGTCGTCTGGATGG
TFAM	CCGGCAGAAACGCCTAAAGA	ATCCTTAGCCCCCTGGAAGC
β-actin	GCGCAAGTACTCTGTGTGGA	CATCGTACTCCTGCTTGCTG

## Data Availability

Not applicable.

## Supplementary Materials

The supporting information can be downloaded at: https://www.mdpi.com/article/10.3390/ijms231810920/s1.
