# Supplementary figures and images for: Anti-Fatigue Effects of Lycium barbarum Polysaccharide and Effervescent Tablets by Regulating Oxidative Stress and Energy Metabolism in Rats
Source: Int J Mol Sci. 2022 Sep 18;23(18):10920. doi: 10.3390/ijms231810920 (PMC9504225; doi:10.3390/ijms231810920)

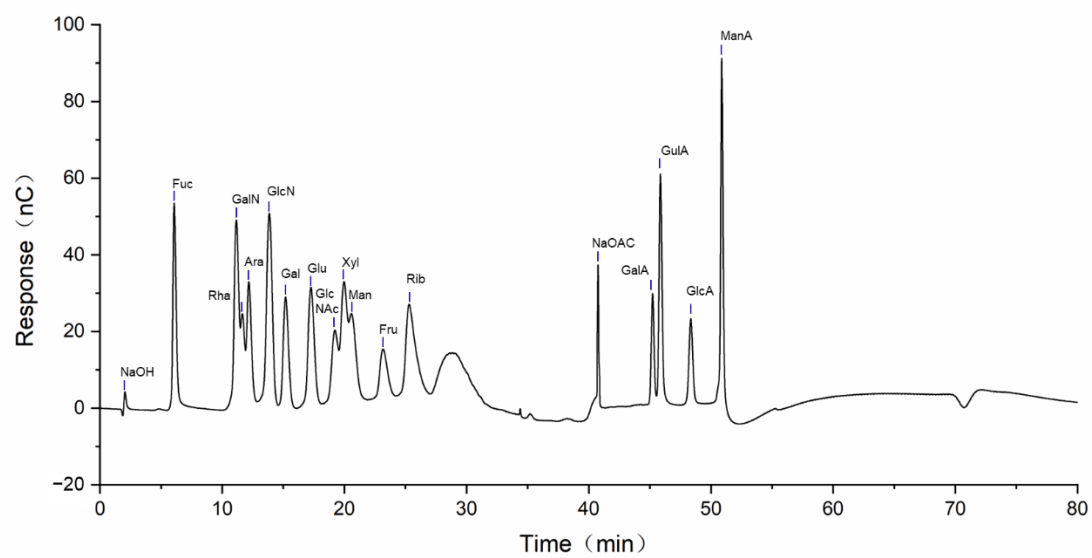

**Figure S1.** Ion chromatogram of 16 monosaccharide standards

Supplement: Supplementary file 1 [file ijms-23-10920-s001.zip › ijms-1904321-supplementary.pdf]
